# Dinaciclib as an effective pan-cyclin dependent kinase inhibitor in platinum resistant ovarian cancer

**DOI:** 10.3389/fonc.2022.1014280

**Published:** 2022-11-25

**Authors:** David Howard, David James, Jezabel Garcia-Parra, Belen Pan-Castillo, Jenny Worthington, Nicole Williams, Zoe Coombes, Sophie Colleen Rees, Kerryn Lutchman-Singh, Lewis W. Francis, Paul Rees, Lavinia Margarit, R. Steven Conlan, Deyarina Gonzalez

**Affiliations:** ^1^ Reproductive Biology and Gynaecology (RBGO) Group, Medical School, Swansea University, Swansea, United Kingdom; ^2^ Axis Bioservices Ltd, Coleraine, United Kingdom; ^3^ Department of Obstetrics and Gynaecology, Princess of Wales Hospital, Cwm Taf Morgannwg University Health Board, Bridgend, United Kingdom; ^4^ Department of Gynaecology Oncology, Singleton Hospital, Swansea Bay University Health Board, Swansea, United Kingdom; ^5^ College of Engineering, Swansea University, Swansea, United Kingdom

**Keywords:** ovarian cancer, resistance, cyclin dependent kinase inhibitor, dinaciclib, cisplatin, platinum, refractory, flavopiridol

## Abstract

**Background:**

Ovarian cancer (OC) is amongst the most lethal of common cancers in women. Lacking in specific symptoms in the early stages, OC is predominantly diagnosed late when the disease has undergone metastatic spread and chemotherapy is relied on to prolong life. Platinum-based therapies are preferred and although many tumors respond initially, the emergence of platinum-resistance occurs in the majority of cases after which prognosis is very poor. Upregulation of DNA damage pathways is a common feature of platinum resistance in OC with cyclin dependent kinases (CDKs) serving as key regulators of this process and suggesting that CDK inhibitors (CDKis) could be effective tools in the treatment of platinum resistant and refractory OC.

**Aim:**

The aim of this study was to evaluate the efficacy of CDKis in platinum resistant OC models and serve as a predictor of potential clinical utility.

**Methods:**

The efficacy of CDKi, dinaciclib, was determined in wildtype and platinum resistant cell line pairs representing different OC subtypes. In addition, dinaciclib was evaluated in primary cells isolated from platinum-sensitive and platinum-refractory tumors to increase the clinical relevance of the study.

**Results and conclusions:**

Dinaciclib proved highly efficacious in OC cell lines and primary cells, which were over a thousand-fold more sensitive to the CDKi than to cisplatin. Furthermore, cisplatin resistance in these cells did not influence sensitivity to dinaciclib and the two drugs combined additively in both platinum-sensitive and platinum-resistant OC cells suggesting a potential role for pan-CDKis (CDKis targeting multiple CDKs), such as dinaciclib, in the treatment of advanced and platinum-resistant OC.

## Introduction

Ovarian cancer (OC) is the 9th most common cancer in women and the 8th most deadly with the latest global figures reporting it accounted for 6.6% of all annual cancer diagnoses in women and 185,000 deaths in 2018 (1). The 5-year survival rate for OC globally currently ranges between 30-40% and in the United States is the 14th lowest survival rate amongst the top 15 most common cancers (1, 2). The principal factors attributed to this high lethality is the disease’s late stage of presentation (80% of OC diagnoses are for stage III and IV cancers) and the development of chemo-resistance (3).

Typical treatment of OC involves surgery and platinum-based chemotherapy, however 80% of cancers are either refractory to platinum treatment, or respond initially, but go on to develop platinum resistance, at which point the disease is typically incurable (4). Inhibition of Poly-ADP-ribose polymerase (PARP) proteins, through inhibitors, such as olaparib, veliparib and niraparib have been shown to delay disease progression in patients with tumors harboring homologous recombination (HR) deficiencies, such as mutations in *BRCA* genes. PARP proteins are involved in single-strand DNA repair and their inhibition leads to an accumulation of single-strand DNA breaks causing synthetic lethality in tumors with existing DNA repair deficiencies (5). However, PARPis are less efficacious in tumors with HR, which represent approximately 50-59% of cases (6). Mutations and epigenetic modifications, which confer PARPi resistance including reversion mutations in HR genes, such as *BRCA1/2*, upregulation of *BRCA1* and downregulation of negative HR regulator, *EMI1*, have all been similarly identified as drivers of platinum resistance (7–10). Undoubtedly it is as a consequence of the mechanistic similarities between platinum and PARPi resistance, that PARPis have proven less effective against cisplatin resistant OC in the clinic (11, 12). Alternative therapies are therefore sorely required to prolong life in patients with advanced and cisplatin resistant OC.

Cyclin dependent kinase (CDKs) are integral to key cellular activities including cell cycle progression, regulation of transcription, DNA repair and DNA replication (13). As these processes tend to be dysregulated in cancer, targeting CDKs through CDK inhibitors (CDKis) may be a promising treatment strategy. Numerous CDKis are currently undergoing clinical and preclinical evaluation for a range of cancers (14) with CDK4/6 specific inhibitors currently in use for hormone receptor-positive metastatic breast cancer (15, 16). In OC, CDKs and the cyclins that regulate their activity are often highly expressed and have been associated with advanced disease, low survivability, recurrence and chemoresistance. For instance, cyclins A and E, activating cyclins of CDKs 1 and 2, are both overexpressed in epithelial ovarian cancer (EOC) where they are associated with platinum resistance and low survivability (17–19), possibly due to the protective roles these CDKs and cyclins play in the DNA damage response (20, 21). CDK5 is another CDK involved in DNA repair, promoting base excision repair (BER) and replication fork repair through upstream regulation of Ape1 (AP endonuclease 1) and *STAT3*, respectively (22). In OC, reduced CDK5 expression has been associated with increased sensitivity to cisplatin and paclitaxel *in vitro* (23, 24). CDKs 7 and 9 are key components of the transcription machinery facilitating the transcription of the majority of genes, including oncogenes that drive proliferation, survival and drug resistance in OC, such as *MYC*, *KRAS* and *HER2* (25–27). High levels of expression of CDK9, CDK7 and CDK7 activator, cyclin H, have all been reported as negative prognostic markers in OC (28–30). CDK12 is involved in the transcription of numerous DNA repair proteins including *BRCA1*, *ATM*, *FANCI*, and *FANCD2* (31).

Here we investigated targeting CDKs as an effective strategy against advanced and platinum-resistant OC. We compared the efficacy of dinaciclib, an inhibitor of CDKs 1, 2, 5, 9 and 12 and flavopiridol, a CDK 1, 2, 4 and 7 inhibitor, in metastatic ovarian cancer cell line, SKOV-3. SKOV-3 cells were most sensitive to dinaciclib, which has a bimodal mechanism of action inhibiting CDKs involved in transcription and cell cycle (32). Dinaciclib induced apoptosis and, arrested cell cycle progression in the G2/M phase. In addition, dinaciclib almost completely eliminated RNA polymerase Ser2 phosphorylation reducing *BCL-2* mRNA levels. The utility of dinaciclib in both platinum-sensitive and -resistant OC was evaluated using primary cells isolated from high grade serous (HGS) tumors and cell line models of cisplatin-sensitive and -resistant disease. Importantly, we show that platinum resistance and refractoriness in OC does not influence sensitivity to dinaciclib. We demonstrate for the first time that dinaciclib is equally effective in cisplatin resistant cell lines and cisplatin refractory primary cells compared to cisplatin sensitive cell lines and primary cells, respectively and furthermore that these drugs combine additively in these cells. Together, our results suggest a potential role for the use of pan-CDKis in the treatment of advanced and platinum-resistant/refractory OC.

## Materials and methods

### Cell culture

A2780 and A2780cis cells were purchased from Sigma-Aldrich (St. Louis, Missouri, US), SKOV-3 and OVCAR-3 cells were purchased from the American Type Culture Collection (Manassas, Virginia, US) and SKOV-3cis and OVCAR-3cis were provided by Axis Bioservices, (Coleraine, UK). A2780 and A2780cis cells were cultured with RPMI 1640 supplemented with 10% Fetal Bovine Serum (FBS), and 1% penicillin-streptomycin (all from Gibco by ThermoFisher, Waltham, Massachusetts, US). OVCAR-3 and OVCAR-3cis cells were cultured with RPMI 1640 supplemented with 20% FBS, 1% penicillin-streptomycin and human insulin (Sigma-Aldrich) to a final concentration of 10 µg/ml. SKOV-3 and SKOV-3cis cells were cultured with McCoys 5A Medium + L-glutamine (Gibco by ThermoFisher) supplemented with 10% FBS, and 1% penicillin-streptomycin. During alternate cell passages, media for cisplatin-resistant cell lines was additionally supplemented with cisplatin at final concentrations of 3 µM for SKOV-3cis, 1.5 µM for OVCAR-3cis and 1 µM for A2780cis cells, respectively.

### Isolation and expansion of patient derived tumor cells

Ovarian tumor cells were isolated and propagated from tumor biopsies obtained from consented patients under ethical approval provided from the Local Ethics Committee (LREC Wales 6, ref 15/WA/0065). Tissue samples were delivered in centrifuge tubes in DMEM F-12 media (Gibco by ThermoFisher). On the day of receipt, tissue samples were gently washed in PBS and then transferred to a petri dish where they were disrupted through persistent chopping with a scalpel. Enzymatic digestion was performed on the disrupted tissue through addition of Collagenase I from Clostridium histolyticum (Gibco by ThermoFisher, CAS No. 9001-12-1) dissolved in a primary cell media to a final concentration of 2 mg/ml, henceforth referred to as primary media, comprising MCDB 105 (Sigma-Aldrich) and Medium 199 (Gibco by ThermoFisher) in a 1:1 ratio. Samples were then transferred to a 37°C, 5% CO2, humidified incubator for thirty minutes, or until the tissue was visibly dissociated. The dissociated tissue samples were transferred to falcon tubes and centrifuged with additional primary media at 300 g for 7 min. The resulting cell pellet was resuspended in primary media supplemented with 20% FBS, 1% penicillin-streptomycin and sodium bicarbonate (Sigma-Aldrich) at a final concentration of 1.8 g/L, henceforth for described as complete primary media. The resuspended cells were transferred to a well of a six well plate and transferred to a 37°C, 5% CO_2_, humidified incubator. Primary cells were expanded and maintained using complete primary media ahead of use in drug treatment experiments.

### Cell viability

Dinaciclib (Selleckchem, Houston, Texas, US) and flavopiridol (Sigma-Aldrich) were dissolved in DMSO (Sigma-Aldrich) to make 10 mM stock solutions and cisplatin (Sigma-Aldrich) was dissolved directly in cell media to a working concentration of 1.66 mM ahead of use. Cell lines and primary cells were seeded in white-walled, 96 well plates (Porvair Sciences, Wrexham, UK) at densities of 500 cells/well for SKOV-3WT/cis, OVCAR-3WT/cis, 2500 cells/well for A2780WT/cis and 1000 cells/well for primary cell cultures. Twenty-four hours following seeding, media was removed and replaced with media containing drug, or vehicle control (DMSO). Treatment media was additionally supplemented with RealTime-Glo™ MT Cell Viability Assay (Promega, Madison, Wisconsin, US) reagents at manufacturer recommended concentrations (1:1000). Treated samples were kept in cell culture incubator and luminescence per well was measured every 24 h in a microplate photometer at 37°C.

### Apoptosis

The RealTime-Glo™ Annexin V Apoptosis Assay (Promega) was used to quantify relative Annexin V levels on SKOV-3 cells. According to manufacturer’s instructions. Cells were seeded with a density of 2000 cells/well in white-walled, 96 well plates (Porvair Sciences) in 100 μl medium and incubated at 37 °C in 5% CO2, humidified air for 24 h. Cells were then treated with, DMSO or dinaciclib at 10, 40 and 80 nM concentrations for 12 and 24 h durations. Luminescence was measured using a microplate photometer. Wells were then washed twice in PBS and a cell viability assay was performed as described above in order to quantify viability per well relative to control wells.

### Cell cycle analysis

Cells were seeded in 6 well plates at densities of 100,000 cells/well and were treated 24 h following with vehicle control, or dinaciclib at 10 and 40 nM concentrations. Cells were then washed in PBS and fixed by incubation with formaldehyde solution, 4% (Sigma-Aldrich). Hoechst 33342 (ThermoFisher Scientific) in PBS at 5 μg/ml was added to the wells and the plates incubated for 4 h in the dark. Cells were washed twice more in PBS and then 3 ml of PBS was added to each well ahead of imaging. Imaging was performed using the IN Cell 2000 (GE Healthcare, Chicago, Illinois, US) high-throughput imaging system with 120 images taken per well using a 20x objective and 450/65-nm emission filter. Images were processed using Cell Profiler 3.0.0 (Broad Institute, Cambridge, Massachusetts, US) (33). Nuclei were segmented by object diameter and Otsu thresholding and the integrated object intensity for each nucleus recorded. A histogram of integrated object intensity vs. number of objects was generated for each sample in Matlab (MathWorks, Natick, Massachusetts, US). G1 and G2/M and S phase peaks were fitted to the nuclear integrated intensity histograms using the Watson Pragmatic algorithm (34) and an integration of peaks yielded the percentages of nuclei per cell cycle phase. Three experimental replicates were performed per sample.

### Immunoblotting

SKOV-3 cells were treated with dinaciclib (10 or 40 nM), or vehicle control for periods of 4 and 20 h. Following a PBS wash, cells were scraped in RIPA buffer (Sigma-Aldrich) supplemented to 1% v/v with each of Proteinase Inhibitor Cocktail 1, Phosphatase Inhibitor Cocktails 2 and 3 (all from Sigma-Aldrich). and transferred into microcentrifuge tubes. Samples were incubated on ice for 30 min with intermittent agitation (vortexing) to insure lysis. Samples were then centrifuged at 20,000 x g for 10 min at 4°C to pellet cell debris and the supernatant containing the protein fraction was retained. Protein samples were quantified using the DC™ Protein Assay (Bio-Rad, Hercules, California, US). Thirty µg of protein was per sample was prepared for electrophoresis by heating at 95°C for 5 min in Laemmli sample buffer containing 5% v/v β-mercaptoethanol (Sigma-Aldrich). Protein samples were then separated through sodium dodecyl polyacrylamide gel electrophoresis (SDS-PAGE) using Mini-PROTEAN^®^TGX™ Precast 4-20% gels (Bio-Rad) and then transferred onto polyvinylidene difluoride (PVDF) membranes using the Trans-Blot^®^ Turbo™ transfer system (Bio-Rad). Membranes were blocked 1 h at RT in 5% bovine serum albumin (BSA) (PAN Biotech, Aidenbach, Germany, USA) in Tris Buffered Saline (TBS) with 0.1% Tween-20 (Sigma-Aldrich) and then probed overnight with anti-Pol II (Ab817) (Abcam, Cambridge, UK), anti-Pol II pSer2 (61083) (Active Motif, Carlsbad, California, United States), and anti-GAPDH (sc25778) (Santa Cruz Biotechnologies, Dallas, Texas, United States) antibodies. Following washes (4x, 5 min) in TBS/Tween-20, membranes were incubated with horse radish peroxidase (HRP) conjugated anti-mouse (NA931V) (GE Healthcare), anti-rabbit (NA934) (GE Healthcare), or anti-rat (sc2032) (Santa Cruz Biotechnologies) secondary antibodies as appropriate for 1 h at RT. Protein bands were detected and imaged using Clarity™ Western Enhanced Chemi Luminescence (ECL) substrate and a ChemiDoc Imager (both supplied by Bio-Rad). Densitometry was performed with ImageLab software (Bio-Rad).

### qPCR

RNA was extracted from control and dinaciclib treated samples using the RNeasy^®^ Mini Kit (Qiagen, Hilden, Germany, Cat. no. 74104) according to the manufacturer protocol from which cDNA was generated using the High Capacity cDNA Reverse Transcriptase Kit (Applied Biosystems by Thermo Fisher Scientific). Reverse transcription reactions were performed using the T100™ Thermal Cycler (Bio-Rad). Samples were analyzed by qPCR in triplicate using the iTaq™ Universal SYBR^®^ Green Supermix (Bio-Rad), run on the CFX96 Real-Time PCR Detection System (Bio-Rad) using previously described primers for RPS18 (forward: 5’-ATTGCCGACAGGATGCAGAA-3’, reverse: 5’-GCTGATCCACATCTGCTGGAA-3’), *MYC* (forward: 5’- CAGCGACTCTGAGGAGGAAC-3’, reverse: 5’- CTGTGAGGAGGTTTGCTGTG-3’), *CCNE1* (forward: 5’- AGAGAACTGTGTCAAGTGGATGG -3’, reverse: 5’- TCTGTGGGTCTGTATGTTGTGTG-3’), *MCL-1* (forward: 5’-TGATCCATGTTTTCAGCGAC-3’, reverse: 5’-AATGGTTCGATGCAGCTTTC-3’), *BCL-2* (forward: 5’-GATGTGATGCCTCTGCGAAG-3’, reverse: 5’-GATGTCTCTGGAATCT-3’) and *BIRC5* (forward: 5’-ACCGCATCTCTACATTCAAG-3’, reverse: 5’-CAAGTCTGGCTCGTTCTC-3’) (32). Calibration curves using serial dilutions of cDNA were plotted and gene expression was quantified by plotting threshold cycle values. Values obtained from reference gene, *RPS18* were used to normalize expression across samples. Relative expression was expressed as the mean fold induction ± standard deviation.

### Statistical analysis

Data distribution was assessed for normality using the Ryan-Joiner and Kolmogorov-Smirnov tests using Minitab v13 (Minitab, State College, Pennsylvania, USA). Non-normally distributed data were analyzed with the nonparametric Kruskal-Wallis test, followed by a Mann-Whitney U test applied *post hoc* to determine statistical significance. Normally distributed data was analyzed by analysis of variance (ANOVA) followed by the Dunnett’s test, or by an unpaired T-test using Prism v6 (Graphpad, San Diego, California, USA) where P < 0.05 was considered significant.

## Results

### Dinaciclib inhibits cell cycle progression and RNA polymerase II phosphorylation

**Figure 1:**
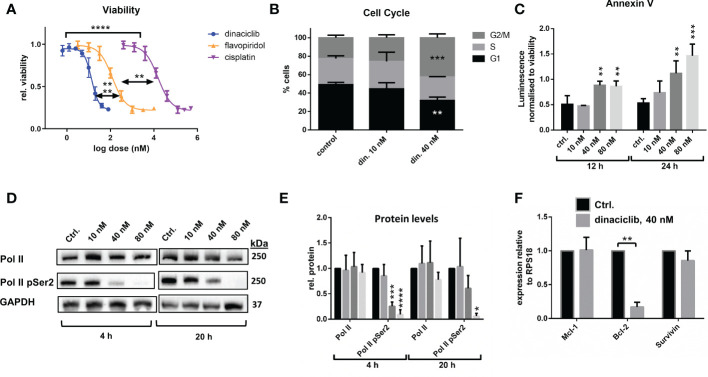
Dinaciclib exhibits a bimodal mechanism in SKOV-3 cells, reducing viability through a combination of cell cycle blockade and apoptosis. **(A)** Dose response curves following 72 h treatments with dinaciclib, flavopiridol and cisplatin in SKOV-3 cells. and its increased potency relative to flavopiridol and cisplatin. Stars indicate statistical significance between LD50 values given in text **(B)** Cell cycle phase was quantified from HCI images stained with Hoescht. **(C)** Annexin V levels in dinaciclib treated SKOV-3 cells, were measured using the luminescence based, RealTime-Glo™ Annexin V Apoptosis Assay (Promega) and normalised to cell viability. **(D)** Representative immunoblots of Pol II and Pol II pSer2 with corresponding for in SKOV-3 cells following 4 and 20 h treatments with dinaciclib, or vehicle control. **(E)** Densitometry measurements for all blots normalised to GAPDH. **(F)** Transcript levels of *MCl-1*, *BCL-2* and *BIRC5* following dinaciclib treatment (40 nM for 24 h) in SKOV-3 cells. Transcript levels were normalised to the reference gene*, RPS18*, and expressed relative to control samples. In all quantitative subfigures, data are expressed as mean of three independent experiments with error bars representing standard deviation. Statistical significance was calculated by ANOVA followed by Dunnett’s test. *p value < 0.05, **p value < 0.01, ***p value < 0.001, ****p value < 0.0001.

The utility of dinaciclib for the treatment of advanced OC was initially investigated using metastatic EOC cell line SKOV-3. Dose-response experiments were performed to compare the anti-proliferative effects of dinaciclib to those of a second pan-CDKi (a CDKi targeting multiple CDKs), flavopiridol, and standard first line therapeutic cisplatin (**Figure 1A**). Dinaciclib proved considerably more potent than both of these drugs with an LD50 of 15 nM compared to 180 nM for flavopiridol and 6 µM for cisplatin. As an inhibitor of CDKs 1 and 2, involved in G2/M and G1/S progression, respectively, it was anticipated that dinaciclib would induce cell cycle blockade. To confirm this, high-content imaging (HCI) of control and dinaciclib treated (24 h) SKOV-3 cells was performed using Hoescht 33342 used as a nucleic acid stain. Cell nuclei were identified and the integrated pixel intensity in the Hoescht channel, corresponding to DNA content, was measured using CellProfiler™ software and plotted against cells to produce DNA content histograms (**Supplementary Figure 1**). Cell cycle distribution was quantified from three replicate experiments (**Figure 1B**). Dinaciclib (40 nM) treatment resulted in an accumulation of cells in the G2/M phase suggesting it is blocking progression through the G2 and or M-phase checkpoints. To test whether the effect on SKOV-3 viability was solely cytostatic, or additionally cytotoxic, cells treated with dinaciclib were evaluated for apoptosis using the RealTime-Glo™ Annexin V Apoptosis Assay (Promega), which detects cell surface exposed phosphatidylserine. Apoptosis was observed in SKOV-3 cell samples treated with dinaciclib at 40 and 80 nM concentrations at 12 and 24 h post-treatment (**Figure 1C**) differentiating pan-CDKi dinaciclib from selective CDK 4/6 inhibitors, which are predominantly cytostatic (35).

Dinaciclib inhibits CDK9, which as part of P-TEFb (positive transcription elongation factor) facilitates transcription elongation by RNA polymerase II (Pol II) through phosphorylation of Ser2 residues on its carboxyl terminal domain (CTD). To determine whether dinaciclib inhibited Pol II phosphorylation in SKOV-3 cells they were treated with vehicle control, or dinaciclib at 10, 40 and 80 nM concentrations for either 4, or 20 h. Levels of total Pol II and of phosphorylated Pol II at Ser2 of the CTD (Pol II pSer2) were quantified in each sample (**Figures 1D and E**). Dinaciclib caused a significant reduction in Pol II pSer2 levels in samples treated with concentrations of 40 nM and above, while levels of total Pol II were not significantly affected. Previous studies have reported a reduction in expression of anti-apoptotic genes caused by dinaciclib, presumably through inhibition of Pol II phosphorylation (36, 37). Here we quantified expression levels of anti-apoptotic genes, *BCL-2* and *MCL-1* and member of the inhibitors of apoptosis (IAP) family, *BIRC5*, by qPCR in cells treated for 24 h with 40 nM dinaciclib (**Figure 1F**). Dinaciclib significantly reduced *BCL-2* transcript levels to 15% compared to controls. Interestingly, *MCL-1* and *BIRC5* mRNA levels were unaffected, suggesting inhibition of P-TEFb affects transcript levels in a gene specific manner. Expression of oncogenes, *MYC* and *CCNE1*, were additionally quantified in SKOV-3 cells following dinaciclib treatment with *MYC* levels greatly reduced while *CCNE1* levels were unaffected (**Supplementary Figure 2**). Together these results indicate that dinaciclib has a bimodal mechanism of action in SKOV-3 cells causing cell cycle blockade and inhibiting Pol II phosphorylation resulting in a gene specific reduction in transcription.

### Dinaciclib is equally effective in cisplatin-sensitive and -resistant cells

**Figure 2:**
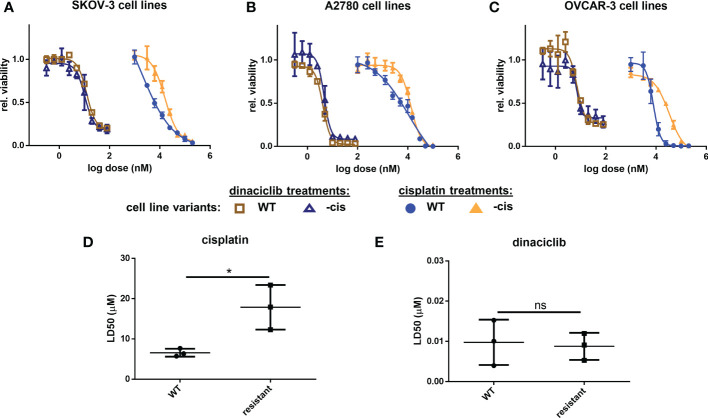
Dinaciclib is effective in cisplatin resistant cell lines. Dose-response experiments for cisplatin and dinaciclib are performed on cisplatin sensitive and resistant cell line variants for **(A)** SKOV-3 and SKOV-3cis **(B)** A2780 and A2780cis and **(C)** OVCAR-3 and OVCAR-3cis cells. Viability is normalised to media controls. LD50s for all cell lines are shown in **(D)** for cisplatin, and **(E)** for dinaciclib. Data are expressed as mean of three independent experiments with error bars representing standard deviation. Statistical significance was calculated by ANOVA followed by Dunnett’s test. *p value < 0.05, ns, not significant.

Platinum-based therapies are the typical first-line treatment of advanced OC, and while often initially effective, the tumors often become resistant, or refractory to these agents (38). When evaluating therapeutics for OC treatment it is therefore essential to consider efficacy against platinum resistant disease. Here, wildtype and cisplatin resistant cell line pairs were used to test the efficacy of dinaciclib in cisplatin sensitive and resistant in vitro models of OC, and to identify any relationship between cisplatin resistance and sensitivity to dinaciclib. Metastatic serous cell line SKOV-3, high grade serous OC cell line OVCAR-3, and endometrioid OC cell line, A2780, were used, with the suffix ‘cis’ (e.g. SKOV-3cis) denoting the cisplatin resistant variants. Dose-response experiments with cisplatin and dinaciclib (**Figures 2A–C**) confirm a difference in sensitivity to cisplatin in the cisplatin sensitive and resistant cell line variants with curves for the cisplatin resistant subpopulations shifted to the right in each plot. As expected, the mean LD50 for cisplatin resistant cell lines is significantly (2.6x) higher than that of the cisplatin sensitive cell line variants (**Figure 2D**). In contrast, there was no significant difference in mean LD50 between cisplatin sensitive (0.010 µM) and cisplatin resistant (0.009 µM) cells treated with dinaciclib (**Figure 2E**). Indeed, LD50 values between individual cisplatin sensitive and resistant variants were nearly identical for dinaciclib: 0.015, 0.004 and 0.010 µM in SKOV-3, A2780 and OVCAR-3 vs 0.012, 0.005 and 0.009 µM in SKOV-3cis, A2780cis and OVCAR-3cis. Significantly, there was no correlation between cisplatin resistance and sensitivity to dinaciclib suggesting mechanisms of cisplatin resistance do not confer resistance to the CDKis.

### Dinaciclib combines additively with cisplatin regardless of cisplatin resistance

**Figure 3:**
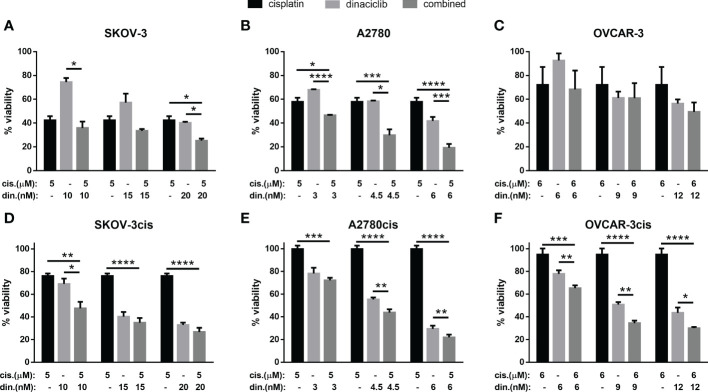
Dinaciclib combines additively with cisplatin in OC cell lines regardless of cisplatin sensitivity. Cells were treated either individually, or in combination with dinaciclib and cisplatin at concentrations reflective of the sensitivities of each cell line to these drugs. Viability was measured after 72 h and normalised to media controls for **(A)** SKOV-3, **(B)** SKOV-3cis, **(C)** A2780, **(D)** A2780cis, **(E)** OVCAR-3 and **(F)** OVCAR-3cis cells. Data are expressed as mean of three independent experiments with error bars representing standard deviation. Statistical significance was calculated by ANOVA followed by Dunnett’s test. *p value < 0.05, **p value < 0.01, ***p value < 0.001, ****p value < 0.0001.

**Figure 4:**
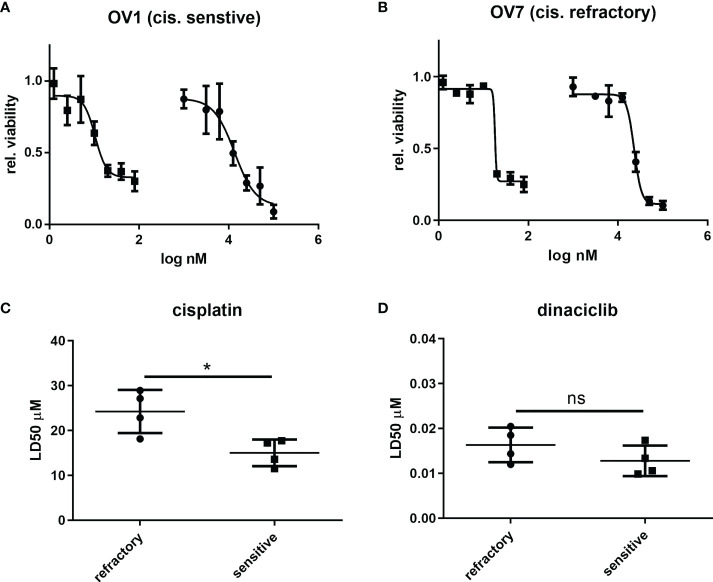
Dinaciclib is equally efficacious in platinum sensitive and refractory patient derived tumour cells. Primary cells were isolated from tumour samples and classified as platinum sensitive (OV1-4), or refractory (OV5-8) based on clinical history (see **Table 1**). Dose response experiments with cisplatin and dinaciclib were performed on the isolated cells with representative curves from platinum sensitive sample and platinum refractory samples shown in **(A)** and **(B)** respectively. Viability is normalised to media controls. LD50s derived from these experiments were compared between platinum sensitive and refractory groups and are shown for cisplatin in **(C)** and dinaciclib in **(D)**. Data are expressed as mean of three independent experiments with error bars representing standard deviation. Statistical significance was evaluated using the significance was calculated using the unpaired t-test. *p value < 0.05, ns, not significant.

With cisplatin currently established as the most effective first line therapy for OC we set out to determine whether dinaciclib could provide and additive advantage over a cisplatin as a monotherapy. Cisplatin sensitive and resistant cell lines were treated for 72 h with cisplatin, dinaciclib, or a combination of the two, with dinaciclib used at the approximated LD25, LD50 and LD75 concentrations, while cisplatin was kept constant at a dose slightly below the LD50 determined for each cisplatin sensitive cells (5 µM, SKOV-3 and A2780; 6 µM, OVCAR-3. Viability assays revealed an additive effect with dinaciclib and cisplatin combination treatments resulting in a significantly lower sample viability than either drug individually (**Figure 3**). Additive effects between dinaciclib and cisplatin were observed in cisplatin sensitive SKOV-3 and A2780, but not cisplatin sensitive OVCAR-3 cells (**Figures 3A–C**). The magnitude of the additive effect was greatest in A2780 cells with the combination of 4.5 nM dinaciclib and 5 µM resulting in an approximate 2-fold decrease in viability compared to either drug alone, and the combination of 6 nM dinaciclib and 5 µM cisplatin resulting in an approximate 3-fold and 2-fold decrease in viability relative to individual cisplatin and dinaciclib treatments. Encouragingly, additive effects between dinaciclib and cisplatin were observed in all cisplatin resistant cell lines (**Figures 4D–F**). Furthermore, additive effects were observed in A2780cis cells at a cisplatin concentration (5 µM) that did not reduce sample viability when not combined with dinaciclib. Given the reduced sensitivity of the resistant cell lines variants to cisplatin, a further set of treatments was performed using cisplatin concentrations at the cisplatin LD50s in these cells (**Supplementary Figure 3**). The additive effects of dinaciclib and cisplatin were maintained in the resistant cells at these higher cisplatin concentrations. Together these results demonstrate that dinaciclib and cisplatin combine additively in both cisplatin sensitive and resistant cells across different ovarian tumor subtypes.

### Dinaciclib is effective as single therapy and in combination with cisplatin in cisplatin sensitive and refractory primary tumor cells

Next, we evaluated whether the efficacy of dinaciclib observed in cisplatin sensitive and resistant cell lines also occurred in cells isolated from tumor biopsies of patients with advanced (stages 3 and 4) OC. Patient biopsies were collected consecutively during interval debulking operations following three cycles of platinum therapy. Tumors were categorized as cisplatin sensitive, or cisplatin refractory on the basis of their response to a platinum-based therapy as assessed by a mid-course CT scan (computerized tomography) where tumors showing a poor, response following chemotherapy were classified as platinum-refractory and those responding well to therapy classified as platinum sensitive. Cells were isolated by mechanical and enzymatic digestion of the biopsy tissue as described in the methods. In total eight tumors were used comprising four platinum sensitive (OV1-4) and four platinum resistant (OV5-8) samples with staging, treatment and recurrence details provided in **Table 1**. Platinum sensitivity was confirmed through dose-response experiments with cisplatin and dinaciclib LD50 values given in **Table 1**. Dose-response curves for cisplatin and dinaciclib for primary cells are shown in **Figures 4A, B** and **Supplementary Figure 4**. The mean LD50s across all primary cell samples for cisplatin and dinaciclib are 19.6 and 0.015 µM, respectively. As expected, the mean cisplatin LD50s are significantly higher in the platinum refractory samples compared to the platinum sensitive group: 24.2 vs 15.0 µM, respectively (**Figure 4C**). In contrast, dinaciclib was equally effective in killing both platinum refractory and sensitive primary cells with similar LD50s reported (0.013 and 0.016 µM, respectively) (**Figure 4D**).

**Table 1:** Cisplatin and Dinaciclib LD50s in platinum sensitive and platinum resistant primary cells.

Sample I.D.	Stage	Chemotherapy	Recurrence	Cisplatin LD50 (µM)	Dinaciclib LD50 (µM)
Platinum sensitive					
OV1	3c	Carboplatin and paclitaxel	none	13.6	0.013
OV2	4a	Carboplatin	none	11.5	0.017
OV3	3c	Carboplatin and paclitaxel	none	9.80	0.017
OV4	3c	Carboplatin and paclitaxel	none	17.7	0.011
Platinum refractory					
OV5	3c	Carboplatin and paclitaxel	3 months	27.1	0.020
OV6	4	Carboplatin and paclitaxel	4 months	28.9	0.012
OV7	3c	Carboplatin and paclitaxel	3 months	22.8	0.018
OV8	3c	Carboplatin and paclitaxel	4 months	18.2	0.014

Samples were grouped based on time-to-recurrence where recurrence within 6 months of treatment with a platinum-based therapy classified as platinum resistant and samples where recurrence occurred beyond 12 months post-treatment classified as platinum sensitive.

**Figure 5:**
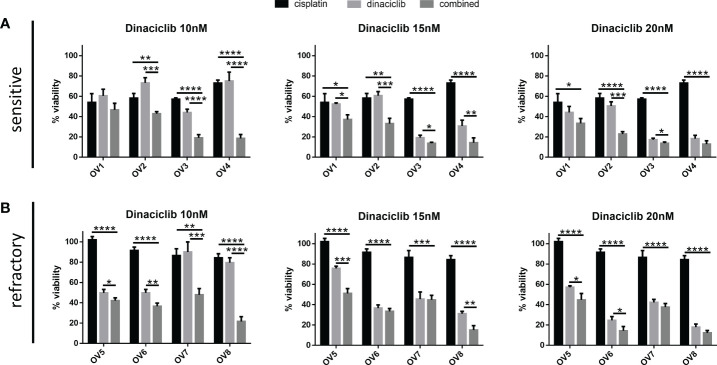
Dinaciclib combines additively with cisplatin in primary samples regardless of cisplatin sensitivity. Patient tumour cells isolated from platinum sensitive and refractory tumours were treated for 72 h either individually, or in combination, with 10 μM cisplatin and dinaciclib at concentrations of 10, 15 and 20 nM. Viability is expressed as a percentage of media controls. Results are grouped by **(A)** platinum sensitive samples (OV1-4) and **(B)** platinum resistant samples (OV5-8). Data are expressed as mean of three independent experiments with error bars representing standard deviation. Statistical significance was calculated by ANOVA followed by Dunnett’s test. *p value < 0.05, **p value < 0.01, ***p value < 0.001, ****p value < 0.0001.

To test whether dinaciclib and cisplatin combined additively in these samples, patient tumor cells were treated individually or in combination with 10 µM cisplatin and dinaciclib at 10, 15 and 20 nM (low, medium and high doses) for 72 h (**Figure 5**). Additive effects were observed across both platinum sensitive and refractory samples. For example, the viability of cells from the cisplatin resistant tumor, OV8, were reduced to 84% of controls following cisplatin treatment, while viability was reduced to only 22% when cisplatin was combined with 10 nM dinaciclib. Interestingly, as with the A280cis cell line, an additive effect between dinaciclib and cisplatin was observed in platinum refractory sample OV5 despite the fact that cisplatin alone had no effect on viability. Consistent with the cell line data, these results highlight the low nM efficacy of dinaciclib in patient tumor cells regardless of platinum sensitivity. Moreover, dinaciclib and cisplatin produce an additive anti-tumor effect in platinum sensitive and refractory primary samples. We report the major observation that dinaciclib remains active in platinum resistant OC in both cisplatin resistant cell lines and cisplatin refractory patient derived tumor cells.

## Discussion

Platinum resistance remains a fundamental challenge in the effective treatment of OC, and despite this challenge relatively few advances have been made in terms of treatment options. Whilst the PARP inhibitors olaparib, veliparib and niraparib delay disease progression in patients harboring *BRCA* mutations, they are limited in terms of the >50% of OC patients whose tumors are proficient in homologous recombination. Furthermore, mutations and epigenetic modifications can result in resistance to PARPi treatment, and PARPis have proven less effective against cisplatin resistant OC in the clinic (39, 40). In contrast, we report the major observation that dinaciclib remains active in platinum resistant OC in both cisplatin resistant cell lines and cisplatin refractory patient derived tumor cells.

To begin, we sought to confirm the bimodal mechanism of dinaciclib, reported elsewhere (32), in SKOV-3 cells and demonstrate that the CDK inhibitor causes cell cycle blockade with an accumulation of cells in G2/M and the inhibition of Ser2 phosphorylation of the Pol II CTD, thus impairing transcription. Other studies have suggested that dinaciclib’s suppression of transcription causes a reduction in the expression of protective, anti-apoptotic genes resulting in cytotoxicity. We therefore decided to test the effects of dinaciclib on the expression of some of these genes and found a significant reduction in *BCL-2*, expression of which has been linked to platinum resistance in resistant ovarian cancer models (41). Interestingly, dinaciclib did not reduce expression of *MCL-1* in SKOV-3 cells as has been previously demonstrated in OC cell line, A2780, and in other tumor types. This discrepancy likely results from the genetic diversity characteristic of OC. For instance, *MCL-1* levels in the wildtype HGS cell line, OVCAR-3, were similarly unaffected by dinaciclib treatment (42).

To evaluate the potential of dinaciclib in platinum resistant and refractory OC, we began by comparing its efficacy across a series of cisplatin sensitive and resistant cell lines. We demonstrate that cisplatin resistance does not correlate with decreased sensitivity to dinaciclib. In all cases, dinaciclib proved equally efficacious with no significant difference in mean LD50s between cisplatin sensitive and resistant cell lines. Moreover, as previously reported (43), we found dinaciclib functioned additively with cisplatin in OC cell lines. Perhaps most significantly, we demonstrate that this additive effect is maintained in cisplatin resistant cell lines, which arguably better reflect the clinical challenge OC poses than the platinum-sensitive *in vitro* models typically used in preclinical OC studies. To enhance the clinical relevance of this study, the efficacy of dinaciclib was investigated in primary cells isolated from ovarian tumor samples. Tumors were classified as either platinum sensitive or refractory based on their response to three rounds of platinum-based chemotherapy as evaluated *via* CT. Cisplatin sensitivity was evaluated in these samples and as expected, the platinum refractory primary cells were significantly less sensitive to cisplatin than the cisplatin sensitive group. In contrast, and consistent with the cell line data, no significant difference in dinaciclib sensitivity was observed with dinaciclib LD50s in the low nM range across platinum sensitive and resistant primary samples. Furthermore, as in the cell lines, dinaciclib combined additively with cisplatin in all primary samples, and specifically in resistant cells where cisplatin alone had no effect on viability. While the results shown here are promising, it is important to consider the importance of the tumor microenvironment (TME), which can reduce drug sensitivity through multiple mechanisms such as decreasing drug penetration release of pro-survival and proliferative factors and immune dampening effects (44). A previous study has reported on the immunogenic effects of dinaciclib in orthotopic and transgenic mouse models of pancreatic ductal adenocarcinoma by reducing the expression of immune checkpoint proteins in the tumor leading to increased immune cell infiltration. Similar studies in platinum resistant OC would be beneficial in evaluating a potential role for dinaciclib in OC therapy.

Currently, CDK4/6 specific inhibitors including ribociclib, palbociclib and abemaciclib are the only class of CDKi approved for clinical use. While their highly targeted mechanism likely contributes to their tolerability it restricts their application to hormone receptor positive, HER2 (human epidermal growth factor receptor 2) negative tumors (45). Furthermore, these drugs are far less efficacious *in vitro* than dinaciclib. Drug IC50 data from the Genomics of Drug Sensitivity in Cancer Project, reports a (geometric) mean IC50 for dinaciclib at 0.07 µM, whereas the mean IC50s for ribociclib and palbociclib are 38.8 and 32.2 µM, respectively (46). However, while the more potent pan-CDKis have proven highly effective in preclinical studies, they have thus far failed to establish themselves in the clinic. Of the first-generation pan-CDKis, flavopiridol has undergone the most extensive clinical testing, but high toxicity led to its eventual discontinuation from development (47). It is likely that, in the case of flavopiridol and other first-generation pan-CDKis, the lack of translation from pre-clinical efficacy to clinical success is a result of a narrow therapeutic window. In response, the developers behind the discovery of dinaciclib, incorporated therapeutic index (the maximum tolerated dose divided by the minimum effective dose) into their compound screening, by measuring responses in A2780 xenografts (48). Using this method, dinaciclib was reported to have a therapeutic index more than 10x greater than that of flavopiridol. Despite this, tolerability has been a concern with thrombocytopenia, neutropenia and gastrointestinal complaints commonly reported (49). While early studies used higher doses of dinaciclib (30-50 mg/m2) (NCT00732810 and NCT01096342), more recent trials have dosed at 14 mg/m2 and below and in combination with other drugs, such as with MK-7965 in types of advanced leukemia (NCT02684617) and MK2206 in pancreatic cancer (NCT01783171). One approach to maximize the anti-tumor effects of dinaciclib while reducing toxicity is to use drug delivery systems such as pro-drugs, nanoparticles, drug-loaded exosomes, or antibody drug conjugates to expand the therapeutic window of dinaciclib and prolong its circulation (50). The pro-drug approach, for instance has led to the re-emergence of flavopiridol. TP-1287 is a phosphate pro-drug of flavopiridol which was shown to have a 75-fold therapeutic index in AML (acute myeloid leukemia) mice xenografts (51) and is currently in a phase 1 clinical trials (NCT03604783). In light of the promising efficacy demonstrated here for platinum resistant advanced OC, expanding the therapeutic window of dinaciclib through drug delivery enhancements such as nanoparticle encapsulation or as an antibody drug conjugate payload is an avenue that should be explored to improve clinical efficacy.

Platinum refractoriness and resistance are hallmarks of advanced OC and therapies that are effective against platinum resistant tumors are urgently needed to improve patient outcomes. Currently the most commonly administered drugs for platinum resistant OC are liposomal doxorubicin, paclitaxel, gemcitabine and paclitaxel (52), however response rates for these drugs in this setting are notoriously low (10-15%) and overall survival with these treatments is just 12 months (53). Our data suggest dinaciclib may be effective against platinum resistant ovarian cancer. Furthermore, combining dinaciclib with platinum therapies in first-line treatment of advanced disease may provide clinical benefit.

## Data availability statement

The raw data supporting the conclusions of this article will be made available by the authors, without undue reservation.

## Ethics statement

The studies involving human participants were reviewed and approved by Local Ethics Committee (LREC) Wales, ref 15/WA/0065. The patients/participants provided their written informed consent to participate in this study.

## Author contributions

DH: acquisition of data; DH and DJ: analysis and interpretation of data; KL-S and LM: provision of clinical samples; DH, LF, DG, LM, JG, BP-C, and RC: contributed to experimental design, procedures, and critical revisions of the manuscript; DH, DG, and RC: drafting the article. All authors made substantial contributions to conception and design of the review and agree to be accountable for all aspects of the work in ensuring that questions related to the accuracy or integrity of any part of the work are appropriately investigated and resolved. All authors contributed to the article and approved the submitted version.

## Funding

This work was funded by Tenovus Cancer Care (grant no. PhD2015/L35), the Life Science National Research Network in Drug Discovery (2016/BPC) and Welsh Government ERDF SMART Expertise 2014-2020 West Wales and the Valleys (2017/COL/001 and 2017/COL/004).

## Acknowledgments

The authors would like to acknowledge and thank the participants of this study and all clinical staff involved for their valuable contribution to ovarian cancer research.

## Conflict of interest

Authors JW and NW were employed by Axis Bioservices Ltd.

The remaining authors declare that the research was conducted in the absence of any commercial or financial relationships that could be construed as a potential conflict of interest.

## Publisher’s note

All claims expressed in this article are solely those of the authors and do not necessarily represent those of their affiliated organizations, or those of the publisher, the editors and the reviewers. Any product that may be evaluated in this article, or claim that may be made by its manufacturer, is not guaranteed or endorsed by the publisher.

## References

[1] Reid BM Permuth JB Sellers TA. Epidemiology of ovarian cancer: a review. Cancer Biol Med (2017) 14:9–32. doi: 10.20892/j.issn.2095-3941.2016.0084 28443200 PMC5365187

[2] Jemal A Ward EM Johnson CJ Cronin KA Ma J Ryerson AB. Annual report to the nation on the status of cancer, 1975-2014, featuring survival. In: Journal of the national cancer institute, vol. 1090. Oxford University Press (2017) 109(9): djx030. doi: 10.1093/jnci/djx030 28376154 PMC5409140

[3] Torre LA Trabert B DeSantis CE Miller KD Samimi G Runowicz CD. Ovarian cancer statistics. CA Cancer J Clin (2018) 68(4):284–96. doi: 10.3322/caac.21456 PMC6621554 29809280

[4] Damia G Broggini M. Platinum resistance in ovarian cancer: Role of DNA repair. Cancers (2019) 11(1):119. doi: 10.3390/cancers11010119 30669514 PMC6357127

[5] Franzese E Centonze S Diana A Carlino F Guerrera LP Di Napoli M. PARP inhibitors in ovarian cancer. Cancer Treat Rev (2019) 73:1–9. doi: 10.1016/j.ctrv.2018.12.002 30543930

[6] Bonadio RRdaCC Fogace RN Miranda VC Diz M del PE. Homologous recombination deficiency in ovarian cancer: a review of its epidemiology and management. Clinics (2018) 73(Suppl 1):e450s. doi: 10.6061/clinics/2018/e450s 30133561 PMC6096977

[7] Kiss RC Xia F Acklin S. Targeting DNA damage response and repair to enhance therapeutic index in cisplatin-based cancer treatment. Int J Mol Sci (2021) 22(15):8199. doi: 10.3390/ijms22158199 34360968 PMC8347825

[8] Li H Liu ZY Wu N Chen YC Cheng Q Wang J. PARP inhibitor resistance: The underlying mechanisms and clinical implications. Mol Cancer (2020) 19(1):1–16. doi: 10.1186/s12943-020-01227-0 32563252 PMC7305609

[9] Moustafa D Abd Elwahed MR Elsaid HH Parvin JD. Modulation of early mitotic inhibitor 1 (EMI1) depletion on the sensitivity of PARP inhibitors in BRCA1 mutated triple-negative breast cancer cells. PLoS One (2021) 16(1):e0235025. doi: 10.1371/journal.pone.0235025 33412559 PMC7790533

[10] Petersen S Wilson AJ Hirst J Roby KF Fadare O Crispens MA. CCNE1 and BRD4 co-amplification in high-grade serous ovarian cancer is associated with poor clinical outcomes. Gynecol Oncol (2020) 157(2):405. doi: 10.1016/j.ygyno.2020.01.038 32044108 PMC7217738

[11] Fong PC Yap TA Boss DS Carden CP Mergui-Roelvink M Gourley C. Poly(ADP)-ribose polymerase inhibition: Frequent durable responses in BRCA carrier ovarian cancer correlating with platinum-free interval. J Clin Oncol (2010) 28(15):2512–9. doi: 10.1200/JCO.2009.26.9589 20406929

[12] Audeh MW Carmichael J Penson RT Friedlander M Powell B Bell-McGuinn KM. Oral poly(ADP-ribose) polymerase inhibitor olaparib in patients with BRCA1 or BRCA2 mutations and recurrent ovarian cancer: A proof-of-concept trial. Lancet (2010) 376(9737):245–51. doi: 10.1016/S0140-6736(10)60893-8 20609468

[13] Roskoski R. Cyclin-dependent protein serine/threonine kinase inhibitors as anticancer drugs. In: Pharmacological research, vol. 139. Elsevier (2019). 139(1):471–88. doi: 10.1016/j.phrs.2018.11.035 30508677

[14] Zhang M Zhang L Hei R Li X Cai H Wu X. CDK inhibitors in cancer therapy, an overview of recent development. Am J Cancer Res (2021) 11(5):1913–35. PMC8167670 34094661

[15] Asghar U Witkiewicz AK Turner NC Knudsen ES. The history and future of targeting cyclin-dependent kinases in cancer therapy. Nat Rev Drug Discovery (2015) 14(2):130–46. doi: 10.1038/nrd4504 PMC4480421 25633797

[16] Sánchez-Martínez C Lallena MJ Sanfeliciano SG de Dios A. Cyclin dependent kinase (CDK) inhibitors as anticancer drugs: Recent advances (2015–2019). In: Bioorganic and medicinal chemistry letters, vol. 29. Elsevier Ltd (2019). p. 126637. doi: 10.1016/j.bmcl.2019.126637 31477350

[17] Taylor-Harding B Aspuria PJ Agadjanian H Cheon DJ Mizuno T Greenberg D. Cyclin E1 and RTK / RAS signaling drive CDK inhibitor resistance *via* activation of E2F and ETS. Oncotarget (2014) 6(2):696–714. doi: 10.18632/oncotarget.2763 PMC4359249 25557169

[18] Topp MD Hartley L Cook M Heong V Boehm E McShane L. Molecular correlates of platinum response in human high-grade serous ovarian cancer patient-derived xenografts. Mol Oncol (2014) 8(3):656–68. doi: 10.1016/j.molonc.2014.01.008 PMC4400120 24560445

[19] Cybulski M Jarosz B Nowakowski A Jeleniewicz W Kutarska E Bednarek W. Cyclin a correlates with YB1, progression and resistance to chemotherapy in human epithelial ovarian cancer. Anticancer Res (2015) 35(3):1715–21. 25750333

[20] Liao H Ji F Geng X Xing M Li W Chen Z. CDK1 promotes nascent DNA synthesis and induces resistance of cancer cells to DNA-damaging therapeutic agents. Oncotarget (2017) 8(53):90662. doi: 10.18632/oncotarget.21730 29207595 PMC5710876

[21] Liu Q Gao J Zhao C Guo Y Wang S Shen F. To control or to be controlled? dual roles of CDK2 in DNA damage and DNA damage response. DNA Repair (Amst) (2020) 85:102702. doi: 10.1016/j.dnarep.2019.102702 31731257

[22] Liu W Li J Song YS Li Y Jia YH Zhao HD. Cdk5 links with DNA damage response and cancer. Mol Cancer (2017) 16(1):60. doi: 10.1186/s12943-017-0611-1 28288624 PMC5348798

[23] Selvendiran K Ahmed S Dayton A Kuppusamy ML Rivera BK Kálai T. HO-3867, a curcumin analog, sensitizes cisplatin-resistant ovarian carcinoma, leading to therapeutic synergy through STAT3 inhibition. Cancer Biol Ther (2011) 12(9):837–45. doi: 10.4161/cbt.12.9.17713 21885917

[24] Zhang S Lu Z Mao W Ahmed AA Yang H Zhou J. CDK5 regulates paclitaxel sensitivity in ovarian cancer cells by modulating AKT activation, p21Cip1- and p27Kip1-mediated G1 cell cycle arrest and apoptosis. PLoS One (2015) 10(7):e0131833. doi: 10.1371/journal.pone.0131833 26146988 PMC4492679

[25] Wrzeszczynski KO Varadan V Byrnes J Lum E Kamalakaran S Levine DA. Identification of tumor suppressors and oncogenes from genomic and epigenetic features in ovarian cancer. PLoS One (2011) 6(12):e28503. doi: 10.1371/journal.pone.0028503 22174824 PMC3234280

[26] Jung MS Russell AJ Kennedy C Gifford AJ Mallitt KA Sivarajasingam S. Clinical importance of myc family oncogene aberrations in epithelial ovarian cancer. JNCI Cancer Spectr (2018) 2(3):pky047. doi: 10.1093/jncics/pky047 31360864 PMC6649713

[27] Reyes-Gonzalez JM Armaiz-Pe~ Na GN Mangala LS Valiyeva F Ivan C Pradeep S. Targeting c-MYC in platinum-resistant ovarian cancer. Molecular Cancer Therapeutics (2015) 14(10):2260–9. doi: 10.1158/1535-7163.MCT-14-0801 PMC4596776 26227489

[28] Kim J Cho YJ Ryu JY Hwang I Han HD Ahn HJ. CDK7 is a reliable prognostic factor and novel therapeutic target in epithelial ovarian cancer. Gynecol Oncol (2020) 156(1):211–21. doi: 10.1016/j.ygyno.2019.11.004 PMC8861881 31776040

[29] Peng C Yang Y Ji L Yang P Yang X Zhang Y. Cyclin h predicts the poor prognosis and promotes the proliferation of ovarian cancer. Cancer Cell Int (2020) 20(1):1–10. doi: 10.1186/s12935-020-01406-5 32694938 PMC7364476

[30] Wang J Dean DC Hornicek FJ Shi H Duan Z. Cyclin-dependent kinase 9 (CDK9) is a novel prognostic marker and therapeutic target in ovarian cancer. FASEB J (2019) 33(5):5990. doi: 10.1096/fj.201801789RR 30726104 PMC6463912

[31] Quereda V Bayle S Vena F Frydman SM Monastyrskyi A Roush WR. Therapeutic targeting of CDK12/CDK13 in triple-negative breast cancer. Cancer Cell (2019) 36(5):545–58.e7. doi: 10.1016/j.ccell.2019.09.004 31668947

[32] Howard D James D Murphy K Garcia-Parra J Pan-Castillo B Rex S. Dinaciclib, a bimodal agent effective against endometrial cancer. Cancers (2021) 13:1135. doi: 10.3390/cancers13051135 33800911 PMC7962054

[33] McQuin C Goodman A Chernyshev V Kamentsky L Cimini BA Karhohs KW. CellProfiler 3.0: Next-generation image processing for biology. PLoS Biol (2018) 16(7):e2005970. doi: 10.1371/journal.pbio.2005970 29969450 PMC6029841

[34] Watson JV Chambers SH Smith PJ. A pragmatic approach to the analysis of DNA histograms with a definable G1 peak. Cytometry (1987) 8(1):1–8. doi: 10.1002/cyto.990080101 3803091

[35] Cretella D Fumarola C Bonelli M Alfieri R La Monica S Digiacomo G. Pre-treatment with the CDK4/6 inhibitor palbociclib improves the efficacy of paclitaxel in TNBC cells. Sci Rep (2019) 9:1. doi: 10.1038/s41598-019-49484-4 31506466 PMC6736958

[36] Saqub H Proetsch-Gugerbauer H Bezrookove V Nosrati M Vaquero EM de Semir D. Dinaciclib, a cyclin-dependent kinase inhibitor, suppresses cholangiocarcinoma growth by targeting CDK2/5/9. Scientific Reports (2020) 10:18489. doi: 10.1038/s41598-020-75578-5 33116269 PMC7595101

[37] Gregory GP Hogg SJ Kats LM Vidacs E Baker AJ Gilan O. CDK9 inhibition by dinaciclib potently suppresses mcl-1 to induce durable apoptotic responses in aggressive MYC-driven b-cell lymphoma *in vivo* . Leukemia (2015) 29(6):1437–41. doi: 10.1038/leu.2015.10 PMC4498453 25578475

[38] Raja FA Chopra N Ledermann JA. Optimal first-line treatment in ovarian cancer. Ann Oncol (2012) Suppl 10:x118–27. doi: 10.1093/annonc/mds315 22987945

[39] Matsumoto K Nishimura M Onoe T Sakai H Urakawa Y Onda T. PARP inhibitors for BRCA wild type ovarian cancer; gene alterations, homologous recombination deficiency and combination therapy. Jpn J Clin Oncol (2019) 49(8):703–7. doi: 10.1093/jjco/hyz090 31242303

[40] Mcmullen M Karakasis K Madariaga A Oza AM. Cancers overcoming platinum and PARP-inhibitor resistance in ovarian cancer. Cancers (2020) 12(6):1607 doi: 10.3390/cancers12061607 32560564 PMC7352566

[41] Xu L Xie Q Qi L Wang C Xu N Liu W. Bcl-2 overexpression reduces cisplatin cytotoxicity by decreasing ER-mitochondrial Ca2+ signaling in SKOV3 cells. Oncol Rep (2018) 39(3):985–92. doi: 10.3892/or.2017.6164 PMC5802038 29286126

[42] Au-yeung G Lang F Azar WJ Mitchell C Jarman KE Lackovic K. Selective targeting of cyclin E1-ampli fi ed high-grade serous ovarian cancer by cyclin- dependent kinase 2 and AKT inhibition. Clinical Cancer Research (2017) 23(7):1862–75. doi: 10.1158/1078-0432.CCR-16-0620 PMC5364079 27663592

[43] Chen XX Xie FF Zhu XJ Lin F Pan SS Gong LH. Cyclin-dependent kinase inhibitor dinaciclib potently synergizes with cisplatin in preclinical models of ovarian cancer. Oncotarget (2015) 6(17):14926–39. doi: 10.18632/oncotarget.3717 PMC4558126 25962959

[44] Giraldo NA Sanchez-Salas R Peske JD Vano Y Becht E Petitprez F. The clinical role of the TME in solid cancer. Br J Cancer (2018) 120:1. doi: 10.1038/s41416-018-0327-z 30413828 PMC6325164

[45] George MA Qureshi S Omene C Toppmeyer DL Ganesan S. Clinical and pharmacologic differences of CDK4/6 inhibitors in breast cancer. Front Oncol (2021) 11:2471. doi: 10.3389/fonc.2021.693104 PMC8313476 34327137

[46] Genomics of Drug Sensitivity in Cancer. Available at: https://www.cancerrxgene.org/.

[47] Xu H Yu S Liu Q Yuan X Mani S Pestell RG. Recent advances of highly selective CDK4/6 inhibitors in breast cancer. J Hematol Oncol (2017) 10(1):97. doi: 10.1186/s13045-017-0467-2 28438180 PMC5404666

[48] Paruch K Dwyer MP Alvarez C Brown C Chan T-Y Doll RJ. Discovery of dinaciclib ( SCH 727965 ) : A potent and selective inhibitor of cyclin-dependent kinases. ACS Medicinal Chemistry Letters (2010) 1(5):204–8. doi: 10.1021/ml100051d PMC4007794 24900195

[49] Kumar SK Laplant B Chng WJ Zonder J Callander N Fonseca R. Dinaciclib , a novel CDK inhibitor , demonstrates encouraging single-agent activity in patients with relapsed multiple myeloma. Blood (2015) 125(3):443–8. doi: 10.1182/blood-2014-05-573741 PMC4296007 25395429

[50] Howard D Garcia-Parra J Healey GD Amakiri C Margarit L Francis LW. Antibody-drug conjugates and other nanomedicines: The frontier of gynaecological cancer treatment. Interface Focus (2016) 6(6):20160054. doi: 10.1098/rsfs.2016.0054 27920893 PMC5071815

[51] Kim W Haws H Peterson P Whatcott CJ Weitman S Warner SL. Abstract 5133: TP-1287, an oral prodrug of the cyclin-dependent kinase-9 inhibitor alvocidib. In: Proceedings of the American Association for Cancer Research Annual Meeting 2017. (2017) Washington, DC. Philadelphia (PA): AACR; Cancer Res 77(13 Suppl):Abstract nr 5133. doi: 10.1158/1538-7445.AM2017-5133

[52] Davis A Tinker AV Friedlander M. Platinum resistant” ovarian cancer: What is it, who to treat and how to measure benefit? In: Gynecologic oncology, vol. Vol. 133. Academic Press Inc (2014). p. 624–31. doi: 10.1182/blood-2014-05-573741 24607285

[53] Pujade-Lauraine E Banerjee S Pignata S. Management of platinum-resistant, relapsed epithelial ovarian cancer and new drug perspectives. J Clin Oncol Am Soc Clin Oncol (2019) 37:2437–48. doi: 10.1200/JCO.19.00194 31403868

