# Supplementary figures and images for: Dinaciclib as an effective pan-cyclin dependent kinase inhibitor in platinum resistant ovarian cancer
Source: Front Oncol. 2022 Nov 25;12:1014280. doi: 10.3389/fonc.2022.1014280 (PMC9732436; doi:10.3389/fonc.2022.1014280)

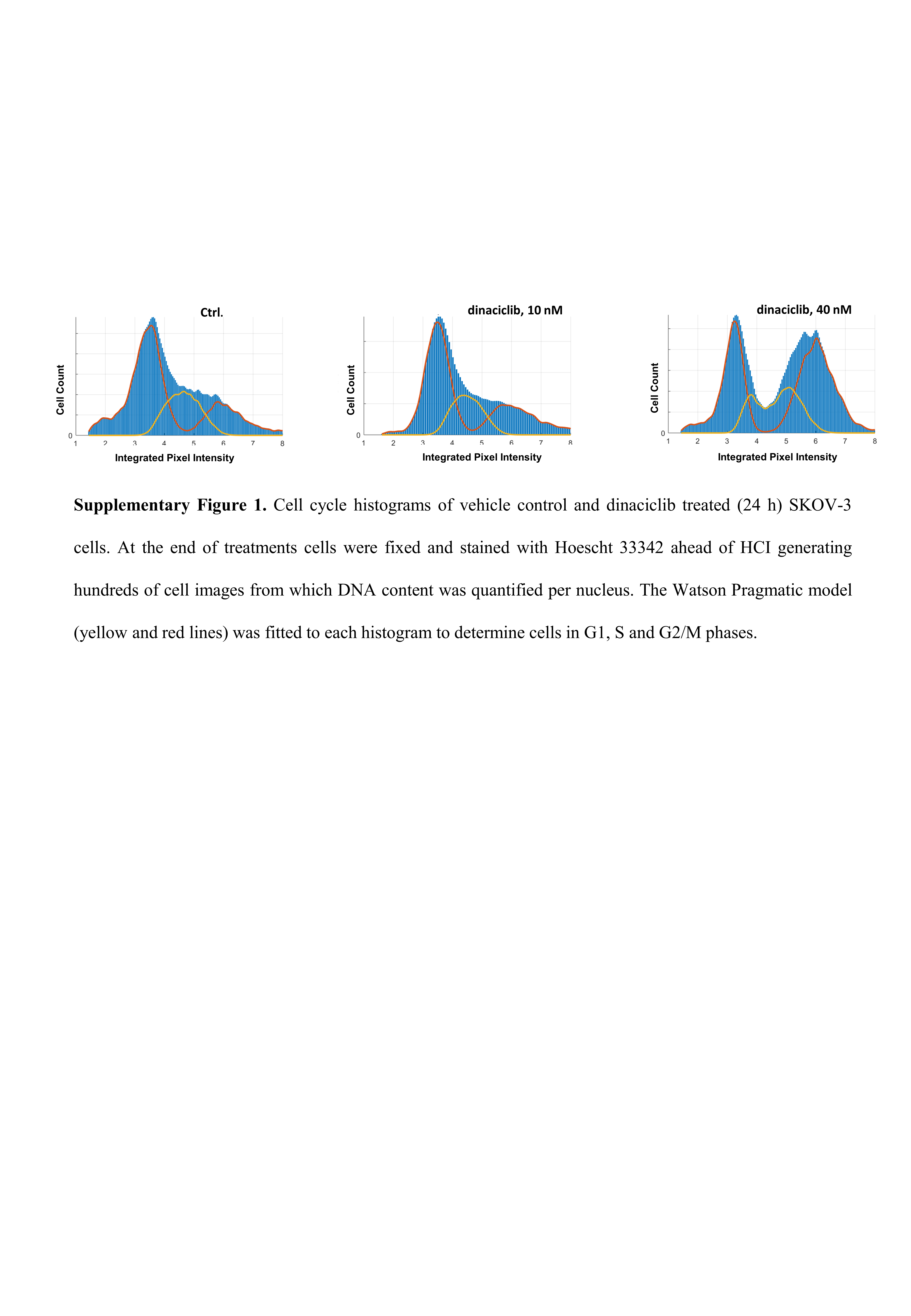

Supplement: Supplementary file 1 [file Image_1.tif]

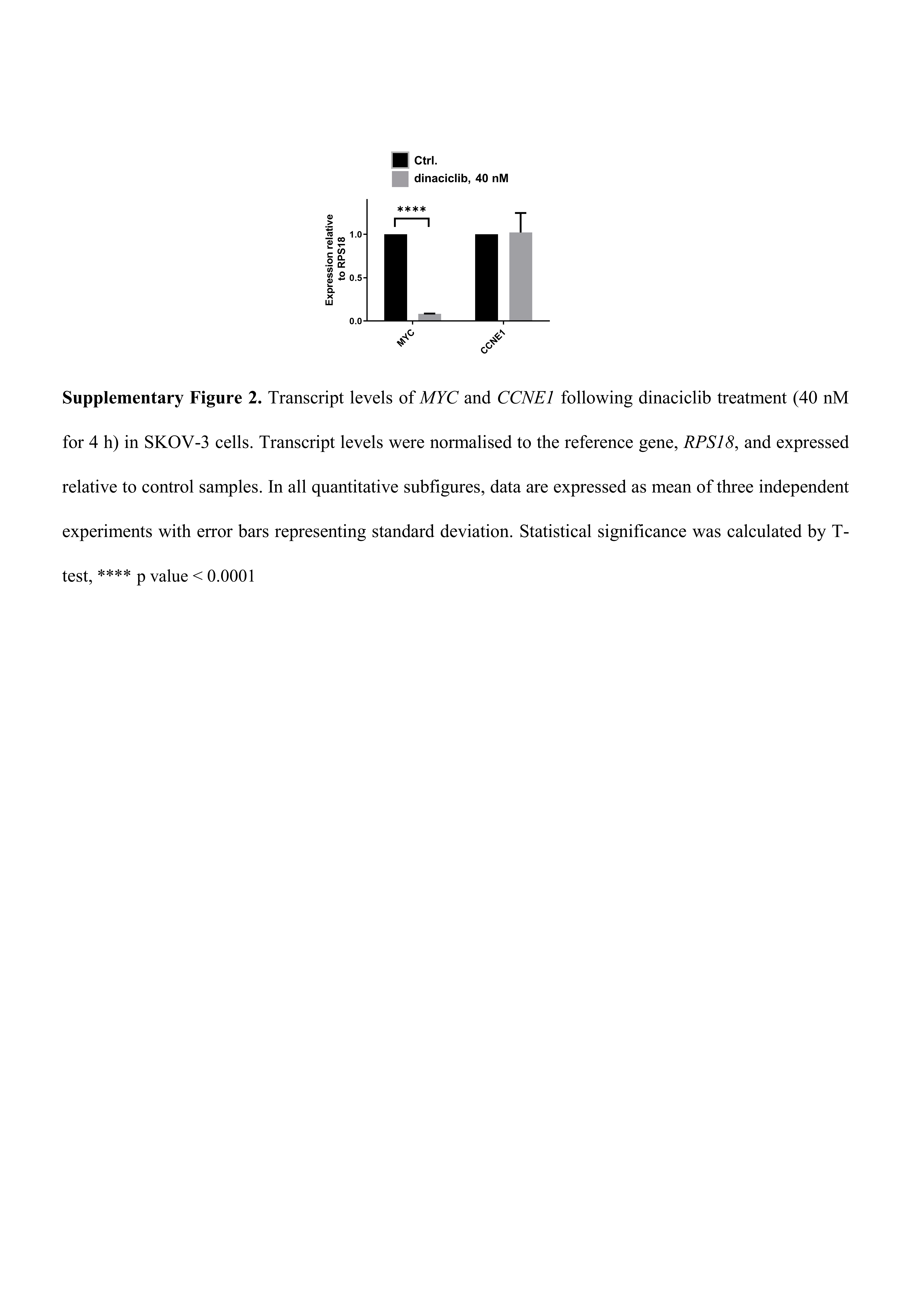

Supplement: Supplementary file 2 [file Image_2.tif]

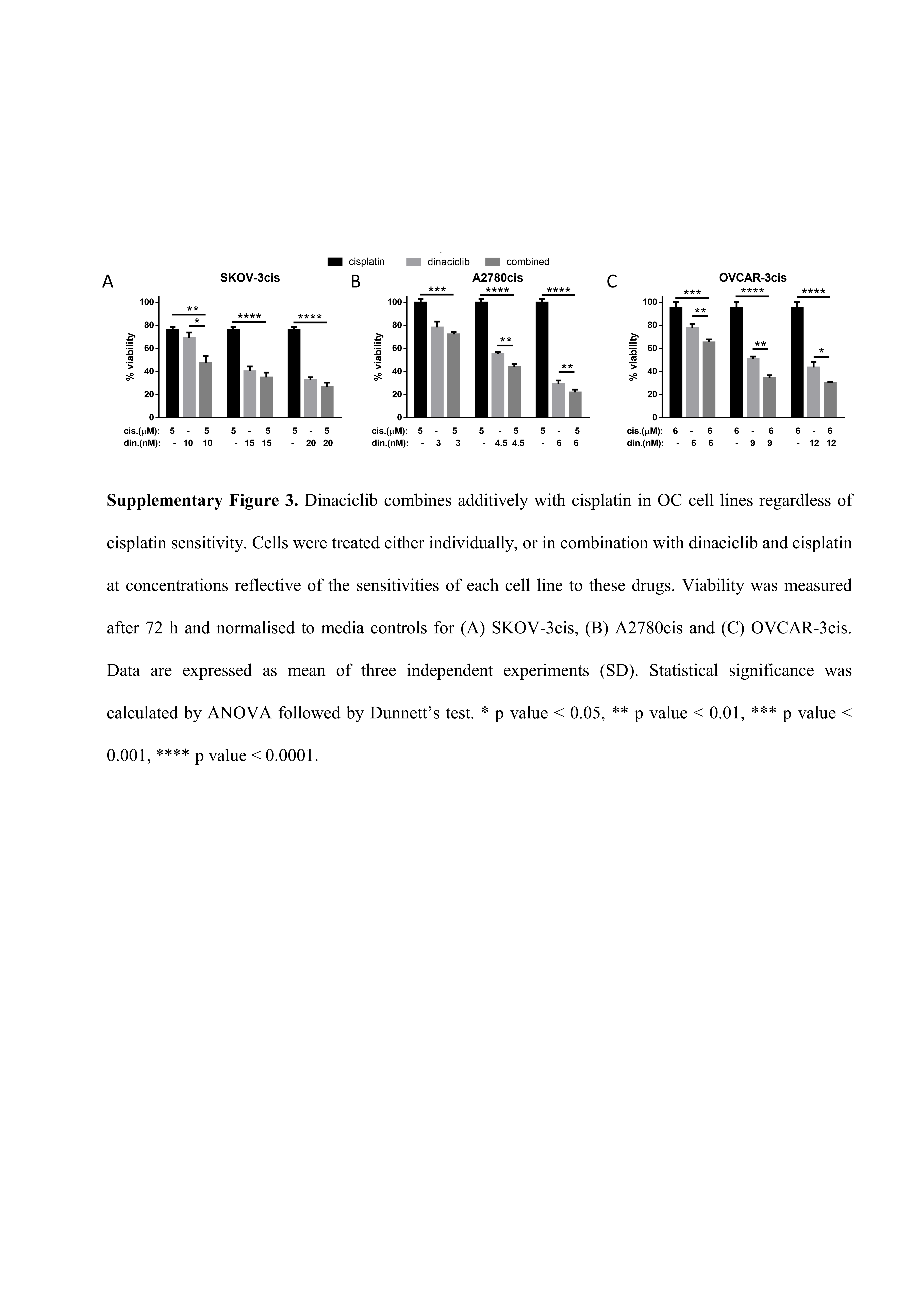

Supplement: Supplementary file 3 [file Image_3.tif]

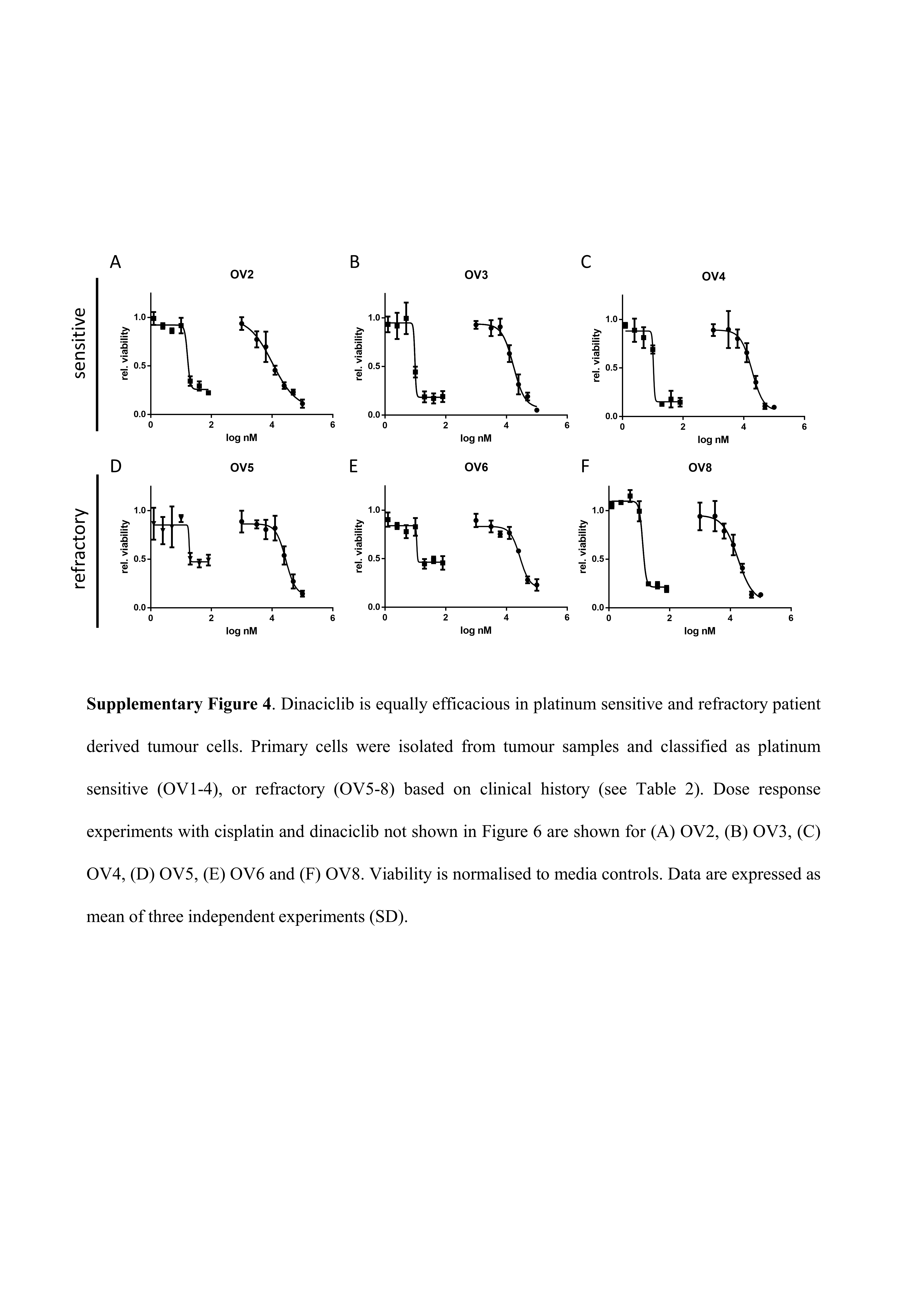

Supplement: Supplementary file 4 [file Image_4.tif]
